# British American Tobacco ghost-wrote reports on tobacco advertising bans by the International Advertising Association and J J Boddewyn

**DOI:** 10.1136/tc.2008.025148

**Published:** 2008-03-18

**Authors:** R M Davis

## Abstract

In 1983 and 1986, the International Advertising Association (IAA) published an original version and then a revision of a report entitled “Tobacco Advertising Bans and Consumption in 16 Countries,” which were edited by J J Boddewyn, a marketing professor. The reports concluded that tobacco advertising bans have not been accompanied by any significant reduction in tobacco consumption. Opponents of tobacco advertising restrictions trumpeted the IAA reports in print materials, media communications and legislative hearings during the 1980s and beyond. A new analysis of tobacco industry documents and transcripts of tobacco litigation testimony reveals that British American Tobacco ghost-wrote the IAA reports and that the Tobacco Institute (the trade association then representing the major US cigarette manufacturers) helped to arrange for Boddewyn to present the findings to the US Congress and the media. Further research on tobacco industry documents and tobacco litigation transcripts should assess whether tobacco industry sources were responsible for ghost-writing other studies favourable to the industry.

In October 1983 and April 1986, the International Advertising Association (IAA) published an original version and then a revision of a report entitled “Tobacco Advertising Bans and Consumption in 16 Countries”.^1^ ^2^ It presented data on trends in cigarette consumption in eight Centrally Planned Economies that had no tobacco advertising, and in eight Free Market Economies, seven of which had banned tobacco advertising. The report concluded “There is no evidence from those countries where tobacco advertising has been banned, that the ban has been accompanied by any significant reduction in overall consumption, per-capita consumption or the incidence of smoking.”^1^ ^2^

The cover of the report (fig 1) attributes the “Introduction and Editing” to Professor J J Boddewyn, a professor of marketing/international business at Baruch College, City University of New York. The preface to the 1986 edition states “The I.A.A. selected Professor Boddewyn to write the Introduction and edit this report because of his numerous works for the I.A.A. on advertising regulations.”^2^ The preface goes on to state “The report was prepared by and from industry sources, using data assembled from official and trade organizations.”^2^ However, the report provides no further information on who conducted the analyses and wrote the text. Thus, the IAA publication links the 16-country study to the IAA itself, to Boddewyn and to “industry sources”, but it is unclear whether “industry” refers to the advertising or tobacco industry.

**Figure 1:**
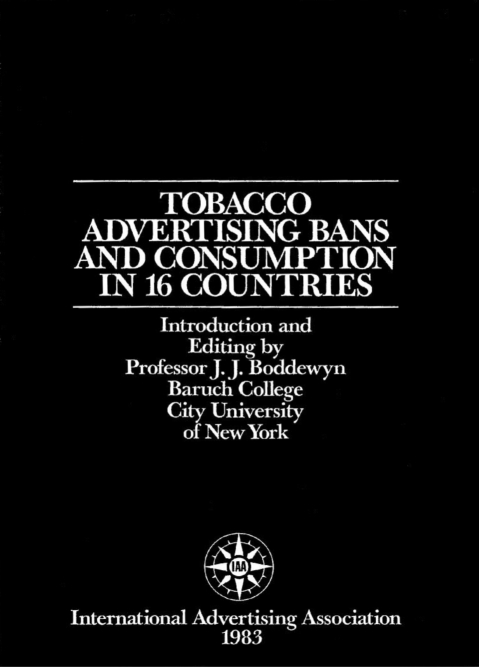
The cover of the International Advertising Association’s 1983 report on tobacco advertising bans and consumption in 16 countries, which was ghost-written by British American Tobacco.

A connection between Boddewyn and the tobacco industry has been known for many years, but a new analysis of tobacco industry documents and transcripts of tobacco litigation testimony reveals a more complete picture of their association. In fact, it shows that British American Tobacco (BAT) ghost-wrote the IAA’s reports on the 16-country study and that the Tobacco Institute (the trade association then representing the major US cigarette manufacturers) helped to arrange for Boddewyn to present the findings to the US Congress and the media.

## SOURCES OF DATA

Relevant transcripts of tobacco litigation testimony were found during a study of testimony on tobacco advertising and promotion as part of the Tobacco Deposition and Trial Testimony Archive (Tobacco DATTA) project. Details on the methods used in that project and study are available elsewhere.^3^ ^4^ In addition, the Legacy Tobacco Documents Library (http://legacy.library.ucsf.edu/) was searched in May 2007 using “Boddewyn” as the search term, and a list of 3304 documents was returned. Only the first 200 of these documents were reviewed because most of the last 50 of these documents were duplicates of earlier documents or were not relevant to this inquiry. More selective searches of the Legacy collection were conducted to find documents related to those identified in the initial search.

## GHOST-WRITING THE IAA REPORTS

In the transcript of testimony by Michael Waterson (a tobacco industry consultant) in litigation over Canada’s national tobacco control act of 1997,^5^ the following exchange occurs with Maurice Regnier, an attorney representing the Canadian Justice Department:

**Question (Regnier):** “When we were reviewing … the document by Infotab, which was not filed [in the court’s public record], you mentioned that … you had knowledge of a work by Boddewyn titled ‘*Tobacco Advertising Bans and Consumption in 16 Countries*’, that’s correct?”

**Answer (Waterson):** “I said I thought I had a memory of it, yes, that’s correct.”

**Q:** “Did you know that this paper by Mr. Boddewyn was in fact ghost-written by Mr. Paul Bingham from British American Tobacco?”

**A:** “I had no idea. I may have seen it.… I had no idea whether one person wrote it or another.”

**Q:** “I would like to show you, Sir, a document that has been filed through Mr. Jean-Paul Blais’ discovery.… It was already filed in the record, My Lord. It is document … ITL-124.… This document is signed by Mr. Paul Bingham. The third paragraph reads:

‘You already have the IAA booklet by Boddewyn, which I ghost-wrote for him in nineteen eighty-six (1986). Although I cannot update this for you instantly, I gave you incidence of smoking numbers, as requested, for some of the countries that had bans.’

Do you have any knowledge, in view of this statement by Mr. Bingham, that Mr. Boddewyn’s booklet was ghost-written by Mr. Bingham?”

**A:** “I have simply … no knowledge of this at all.” (italics in original)

Regnier then asks the court to file the ITL-124 document (enter it into the court’s public record), but Simon Potter, an attorney for the Imperial Tobacco Company, objects on the basis that the document “was filed during a discovery under une ordonnance de confidentialité, pour des raisons commerciales [a confidentiality order, for commercial reasons].”

Although the aforementioned document is not publicly available (Rob Cunningham, Canadian Cancer Society, personal communication, 13 July 2006), other industry documents that are available confirm that Bingham ghost-wrote both the 1983 and 1986 versions of the IAA report. One such document,^6^ from 1983, was in the collection of the Brown & Williamson Tobacco Corporation, formerly a subsidiary of BAT. It is entitled “Report from the Secretariat,” presumably referring to the secretariat of the industry’s International Tobacco Information Centre (INFOTAB).^7^ This document states that “Further meetings have been held and final arrangements made with Professor Jean Boddewyn and the N.Y. office of the I.A.A. for the monograph on advertising bans and their effects to be published as soon as possible.”^6^ This update appears under the heading “Bingham Paper.”

According to an INFOTAB memo^8^ dated 30 January 1986, entitled “16 Bans Booklet”:

“[W]e have just received the updated manuscript from Paul Bingham of BAT Milbank, including figures for 1983 and 1984, which continue to support the basic position.… We are in touch with the IAA and plan to have the updated booklet available just as soon as possible. Our thanks go again to Paul Bingham and BAT for putting together the data. This has proved to be an invaluable item in the advertising debate and much used in our outlets.”

In an apparent handout for a presentation at an INFOTAB workshop held on 12–15 October 1987 in Washington, DC, Boddewyn refers to “the INFOTAB’s study of *Advertising Bans in 16 Countries* (published by the International Advertising Association).”^9^

In summary, the 1983 and 1986 reports were ghost-written by Paul Bingham of BAT, published by IAA, edited by Boddewyn, and then attributed by Boddewyn to INFOTAB.

## PUBLICISING THE IAA REPORTS

Opponents of tobacco advertising restrictions trumpeted the IAA reports in print materials, media communications and legislative hearings during the 1980s and beyond. For instance, Boddewyn highlighted the results of the 16-country study in hearings before the US House of Representatives in 1986, 1987 and 1989.^10^^–^12 A Tobacco Institute (TI) memorandum distributed internally before the 1989 hearing indicates that the TI requested that Boddewyn and three other university-based “experts” be invited to testify.^13^ Another internal TI memo—distributed after the hearing—summarises the hearing, mentions Boddewyn’s testimony about the 16-country study and indicates that Boddewyn was one of two witnesses on the fourth panel “testifying on behalf of the industry.”^14^ The memo also states that media coverage of the hearing was “moderate” and that “Tobacco Institute public affairs staff was on hand to promote the industry’s positions with the press, and to facilitate interviews with experts.”^14^

Interestingly, in the “Editor’s introduction” in the 1986 edition of the IAA report, Boddewyn cites the publicity surrounding the original (1983) report as a reason for revising and republishing it: “Since the first edition of this study has elicited much interest and has been widely used in recent discussions and governmental hearings, it was thought appropriate to update it to 1984.”^2^

## CONNECTIONS BETWEEN BODDEWYN AND THE TOBACCO INDUSTRY

Boddewyn did not disclose his personal affiliation with the tobacco industry in the IAA’s 1983 and 1986 reports. However, in a 1989 report from the IAA on a study of children’s self-reported reasons for starting to smoke, Boddewyn did acknowledge that he “was asked by INFOTAB, the tobacco industry’s international information centre, and by the International Advertising Association, to edit this report, and to comment on its validity and significance”.^15^ ^i^ In an article published in 1989 in the *British Journal of Addiction* (now called *Addiction*), Boddewyn confessed “I am biased because I have served as a paid expert witness for the tobacco industry in the United States and Canada.”^18^

A search of documents in the Legacy Tobacco Documents Library (http://legacy.library.ucsf.edu/) shows myriad connections between Boddewyn and the tobacco industry. For example, an internal Philip Morris memorandum requests a $6000 payment to Boddewyn for the preparation of a 25–50-page report that would, among other things, “discuss and refute claims that Marlboro marketing activities are aimed at children.”^19^ That memo also requested “Payment of expenses for attending hearings, etc.” For a fee of $10 000 plus travel expenses (including business-class airfare), Boddewyn agreed to testify before the Social Services Select Committee for the Tobacco Institute of New Zealand. That institute wrote to Philip Morris and thanked the company for agreeing to cover that funding, but indicated that Boddewyn “will be in New Zealand at the invitation of the Newpaper Publishers Association of New Zealand.”^20^ ^21^

Joossens cited a paper presented by Boddewyn in March 1988 to Belgian journalists who were invited to Washington, DC, by the Belgian tobacco industry.^22^ The Secretary General of INFOTAB claimed to have arranged for Boddewyn to be a tobacco industry witness at a January 1985 hearing in Hong Kong on proposed restrictions of tobacco advertising in broadcast media.^23^ Boddewyn reportedly also attended BAT media seminars in 1992 and 1993 in Bali, South Africa and Sri Lanka.^24^

On 14–15 September 1987, the US Tobacco Institute held its 15th College of Tobacco Knowledge in Washington, DC, for “our friends in the tobacco family.” Memoranda for this event indicated that the college “will focus on the issues most important to the industry … with the Public Affairs Division issues team coordinating sessions that highlight our most effective arguments, experts, allies and other resources.” Registration was limited to 75 people. Boddewyn was one of four speakers listed on the agenda for the session on “Advertising Restrictions,” which was moderated by Frederick Panzer, vice president of the Tobacco Institute.^25^^–^27 Boddewyn was a speaker on a similar panel at the institute’s September 1988 College of Tobacco Knowledge,^28^ ^29^ whose attendees included a diverse mix of people from cigarette companies, the Tobacco Institute and advertising and public relations firms.^30^

The Tobacco Institute’s collection of documents includes a copy of a letter from the editor of the *British Journal of Addiction* to Boddewyn, about peer-review comments for a manuscript that Boddewyn had submitted to the journal.^31^ A manuscript written by Boddewyn, perhaps the same as that mentioned in the preceding sentence, is also found in the institute’s files, with a scribbled note on the cover page from “Jean” (Boddewyn) to “Fred” (presumable Panzer).^32^ The existence of these notes and materials in the institute’s files suggests collaboration between Boddewyn and the tobacco industry in his publication activities. Confirmation of that collaboration comes from a handwritten note from Boddewyn to Jean Besques (of Philip Morris in Lausanne, Switzerland), asking for suggestions on revisions to the manuscript he submitted to the *British Journal of Addiction*.^33^

In a briefing paper based on material in the Minnesota depository of tobacco industry documents, Hirschhorn presented other evidence indicating a connection between Boddewyn and the tobacco industry.^24^

## FLAWS IN THE IAA REPORTS

The IAA study published in 1983 and 1986 presented descriptive data on tobacco consumption for 16 countries, all but one of which prohibited tobacco advertising. The study found that “advertising bans have not been followed by significant changes in tobacco consumption.” Boddewyn, in his “Editor’s introduction,” concluded that tobacco advertising bans are “deplorable” because “they appear to be unrelated, in the short or medium term, to overall tobacco consumption” and “they also tend to prevent or hamper the spreading of information about new features such as filtered and lower tar cigarettes.”^1^

A major flaw in Boddewyn’s reasoning is that tobacco consumption might have been higher in these countries if tobacco advertising had been allowed—a possibility acknowledged by Boddewyn in his testimony before Congress in 1986.^10^ But in that same testimony, he conjures up the “straw man” premise that tobacco advertising is the only factor purported to affect tobacco use in the population, and then he cites IAA data to knock down that straw man. Boddewyn, for example, argues repeatedly that “factors other than advertising are at work”.^10^ However, experts in tobacco tobacco do not argue that advertising is the *only* factor, or even the *main* factor, in determining patterns of tobacco use in the population. Instead, most of them assert that it is *one of several factors* that influence tobacco consumption.

To assess the independent effect of one of those factors on tobacco consumption, studies must be designed so as to take into account other factors associated with tobacco use. The IAA study did not include any other controls on tobacco demand such as tobacco price or income. Because changes in price and income can have a larger effect on tobacco demand than advertising bans, the failure to control for these variables makes it impossible to determine the effect of tobacco advertising bans from the IAA study.^34^

Saffer and Chaloupka, on the other hand, in a study of the effects of tobacco advertising restrictions on tobacco consumption in 22 Organization for Economic Cooperation and Development (OECD) countries, controlled for several correlates of tobacco use including price, income and unemployment.^35^ They found that comprehensive tobacco advertising bans can reduce tobacco consumption, but that partial advertising bans have little or no effect on consumption (because the latter permit a shift of marketing expenditures from “banned” media to “allowed” media).

## CONCLUSIONS

Despite major flaws in the IAA’s 16-country study, it was cited prominently by opponents of tobacco advertising bans in the 1980s and 1990s. Unfortunately BAT’s role in ghost-writing the IAA’s 1983 and 1986 reports was not known during that time, and has only now come to light—more than two decades after their initial publication. Boddewyn’s written statement to Congress in 1989 did acknowledge in a footnote that the 16-country study “was financed by the tobacco industry,”^12^ but that disclosure did not appear in the IAA’s 1983 and 1986 reports. Further research on tobacco industry documents and tobacco litigation transcripts should assess whether tobacco industry sources were responsible for ghost-writing other studies favourable to the industry.

**What this paper adds**

- The 1983 and 1986 reports on the International Advertising Association’s 16-country study on tobacco advertising bans were edited by J J Boddewyn, a marketing professor, and were cited extensively in opposition to tobacco advertising restrictions.
- A connection between Boddewyn and the tobacco industry has been known for many years, but this analysis of tobacco industry documents and transcripts of tobacco litigation testimony provides a more complete picture of their association and reveals that British American Tobacco ghost-wrote the IAA reports.
- The documents also show that the Tobacco Institute (the trade association then representing the major US cigarette manufacturers) helped to arrange for Boddewyn to present the IAA’s findings to the US Congress and the media.

## Footnotes

i The IAA’s 1989 report indicates that children aged 7–16 years were much more likely to attribute their smoking initiation to personal curiousity (“to see what it was like”) and family and peer influences than to cigarette advertising. In his “Editor’s introduction,” Boddewyn comments that this study “breaks new methodological ground … [and] provides strong evidence that advertising plays a negligible role in the initiation of smoking by the young”.^15^ However, as noted in the US Surgeon General’s 1989 report on smoking and health, marketing experts question the validity of smokers’ assessments of the influence of advertising on their smoking behaviour because “conscious response to advertising is deemed to be a poor index of actual response”.^16^ Chapman points out additional flaws in the study, one of which was that the survey questions seemed designed so as to minimise the respondents’ likelihood of identifying cigarette advertising as a factor in their smoking: children in the study were asked to name “the most important reason” why they smoked their *first* cigarette.^17^
